# Green synthesis of surface-passivated carbon dots from the prickly pear cactus as a fluorescent probe for the dual detection of arsenic(iii) and hypochlorite ions from drinking water

**DOI:** 10.1039/c8ra05861j

**Published:** 2018-08-29

**Authors:** K. Radhakrishnan, P. Panneerselvam

**Affiliations:** Department of Chemistry, SRM Institute of Science and Technology Kattankulathur-603 203 Tamil Nadu India panneerselvam.pe@ktr.srmuniv.ac.in panneerchem82@gmail.com +91 96 88 53 88 42

## Abstract

Efforts were made to develop a simple new approach for the green synthesis of surface-passivated carbon dots from edible prickly pear cactus fruit as the carbon source by a one-pot hydrothermal route. Glutathione (GSH) was passivated on the surface of the CDs to form a sensor probe, which exhibited excellent optical properties and water solubility. The prepared sensor was successfully characterized by UV-visible spectrophotometry, fluorescence spectrophotometry, Fourier transform infrared spectroscopy (FT-IR), X-ray diffraction (XRD), scanning electron microscopy (SEM), and transmission electron microscopy (TEM). The simple sensing platform developed by the GSH-CDs was highly sensitive and selective with a “turn-off” fluorescence response for the dual detection of As^3+^ and ClO^−^ ions in drinking water. This sensing system exhibited effective quenching in the presence of As^3+^ and ClO^−^ ions to display the formation of metal complexes and surface interaction with an oxygen functional group. The oxygen-rich GSH-CDs afforded a better selectivity for As^3+^/ClO^−^ ions over other competitive ions. The fluorescence quenching measurement quantified the concentration range as 2–12 nM and 10–90 μM with the lower detection limit of 2.3 nM and 0.016 μM for the detection of As^3+^ and ClO^−^ ions, respectively. Further, we explored the potential applications of this simple, reliable, and cost-effective sensor for the detection of As^3+^/ClO^−^ ions in environmental samples for practical analysis.

## Abstract 2

Efforts were made to develop a simple new approach for the green synthesis of surface-passivated carbon dots from edible prickly pear cactus fruit as the carbon source by a one-pot hydrothermal route.

## Graphical abstract

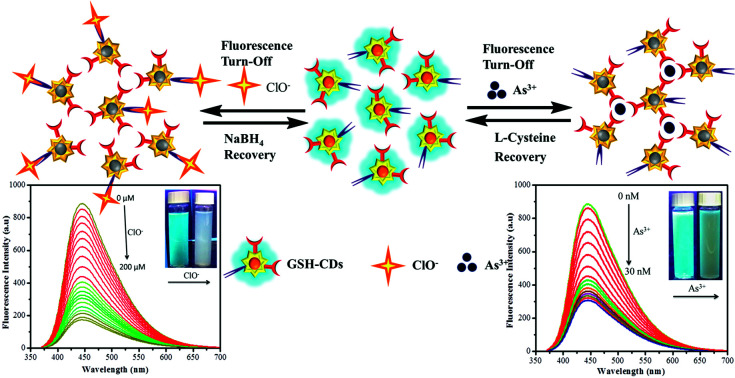

## 1. Introduction

Arsenic (As^3+^) and hypochlorite ions (ClO^−^) have shown an extensive range of poisonous action that can adversely affect the water quality and threaten the public health and the environment. Arsenic contamination has been found to have significantly increased in the environment as a result of industrialization, urbanization, the use of additives in poultry feed, and by agricultural activity.^1–3^ The World Health Organization announced the presence of As^3+^ ions in natural water beyond 10 ppb as a global environmental problem, which has attracted the attention of the research community. The U.S. Environmental Protection Agency and International Association for Research into Cancer have classified As^3+^ as a Group A and Category 1 human carcinogen.^4–6^ As the source of As^3+^/ClO^−^ ions is widespread, they can be easily circulated into ecological processed through our diets.^7–10^ Arsenic accumulation in the human body affects the lungs, kidneys, bladder, and liver and also causes various cancers.^11,12^ Similarly, exposure to hypochlorite ions leads to damage of red blood cells, neuron degeneration, and lung injury.^13–16^ Further, As^3+^/ClO^−^ ions cause a severe health risk of cardiovascular and respiratory diseases.^17–20^ Therefore, it is mandatory and a pressing need for the global research community to develop a user-friendly, selective, sensitive, and reliable method for the detection of As^3+^/ClO^−^ ions.

Many analytical techniques have been developed for the accurate determination of As^3+^/ClO^−^ ions. Trace levels of As^3+^ ions have been quantitatively detected by inductively coupled plasma mass spectroscopy (ICP-MS), inductively coupled plasma atomic emission spectrometry (ICP-AES), atomic fluorescence spectrometry (AFS), and atomic absorption spectrometry (AAS).^21–25^ The common analytical methods used for the accurate detection of As^3+^/ClO^−^ ions are chemosensors, biosensors, and iodometric and polarographic methods.^26–34^ Though these techniques are very qualitative and quantitative, yet they are limited to expensive instrumentation, inconvenient analytical methods, tedious material preparation procedures, and are time consuming. Recently, fluorometric and colorimetric sensors have exhibited an instant response and reusability. Although, they are powerful tools for the potential monitoring of heavy metal ions in real time, the preparation methodology of nanomaterials and functionalization of the utilized organic probe are tedious and limit their wide application. Indeed, a novel fluorescent sensor is still required for the practical detection of As^3+^/ClO^−^ ions in environmental samples.

In the past decade, carbon dots have been emerged as excellent fluorescent probes with unique features. Some of the merits are their eco-friendly nature, water solubility, chemical stability, low toxicity, and biocompatibility. These interesting features of fluorescent CDs have drawn the attention of the scientific community for their application in imaging, diagnosis, catalysis, and energy conversion.^35–39^ Fortunately, carbon dots possess a strong optical response, narrow emission peak, and broad excitation spectra, which give them an obvious advantage over other conventional dyes with challenging properties, like a narrow excitation wave length, broad emission band, and low fluorescence intensity.^40–42^ The prime limitation of CDs is their unsatisfactory sensitivity or fluorescence intensity for the detection of target species based on their poor functionalization.^43^ The sensitivity of the CDs can, however, be apparently improved by surface modification, which comprises surface functionalization and heteroatom doping to afford new classes of CDs. Such surface-passivated CDs have shown improved sensitivity, specificity, an enhancement in their fluorescent efficiency and also improved active sites for better sensing platform performance.^44^ In regards environmental protection, many eco-friendly precursors have been developed to synthesize CQDs from hair, banana juice, pee pollen, and winter melon, *etc.*^45–49^ These sources are highly desirable for achieving a simple, economical, and green synthesis procedure for the preparation of fluorescent CQDs. The application of biomass to prepare carbon nanomaterials has been one strong trend to synthesize a surface-passivated material to improve the selectivity by functionalization and enhanced sensitivity to recognize certain analytical species.

Herein, we report a one-pot synthetic route for the preparation of surface-passivated oxygen-rich GSH-CDs as a multifunctional sensor for the selective and sensitive detection of As^3+^/ClO^−^ ions in drinking water. The GSH-CDs were successfully synthesized from prickly pear cactus as a carbon source and glutathione passivation to render surface-activated carboxylic groups, amine groups, and hydroxyl groups as sensing probes for the sensitive detection of As^3+^/ClO^−^ ions. In addition, the developed green synthesis procedure and sample preparation did not require any tedious conditions. This aspect promotes simplicity, specificity, and also a sensitive response in the pH range of 6 to 8 for the quantitative analysis of the target ions. The quenching of the bright blue emission of the sensing system was used as a signaling unit and its fluorescence intensity was a direct read-out of the specificity in the chelation of As^3+^/ClO^−^ ions. To the best of our knowledge, this was the first attempt to propose surface-passivated fluorescent carbon dots as a fluorescent probe for the sensing of As^3+^/ClO^−^ ions. The sensing mechanism was stated showing the feasibility for dual detection, while the recovery study highlighted the selectivity of the sensor to As^3+^ and ClO^−^ ions.

## 2. Experimental section

### 2.1. Chemicals and materials

The prickly pear cactus was collected from the bare lands near Potheri mount. Tris(hydroxymethyl) methyl amino methane, glutathione, l-cysteine, sodium borohydride (NaBH_4_), and the salts of various metal ions (Ag^+^, K^+^, Ca^2+^, Cu^2+^, Ni^2+^, Ba^2+^, Pb^2+^, Hg^2+^, Cd^2+^, Co^2+^, Fe^2+^, As^3+^,Fe^3+^, Cl^−^, ClO^−^, Br^−^, I^−^, SCN^−^, NO_2_^−^, PO_4_^3−^, H_2_PO_4_^−^, SO_4_^2–^, ONOO^−^, ·OH, and O_2_˙^−^), were purchased from Alfa Aesar. The chemicals and reagents used in this experiment were of analytical grade and were used without further treatment. Ultrapure water (Milli-Q water), drinking water, and tap water were used in preparation of the solutions.

### 2.2. General methods

UV-visible spectroscopic analyses were recorded on a Shimadzu UV-2600 spectrophotometer. The fluorescence spectra of GSH-CDs were measured on a HORIBA JOBIN-YVON Fluoromax-4 spectrofluorometer. FT-IR spectra were measured using an Agilent Resolution Pro FT-IR spectrometer. X-Ray diffraction (XRD) patterns were recorded by PAN analytical X'pert power diffractometer using Cu Kα radiation as the source for excitation. Transmission electron microscopic (TEM) images of the sample were analyzed on a JEOL/JEM-2100 at an operating voltage of 2000 kV. The fluorescence quenching lifetime of the GSH-CDs was examined using a JOBIN-YVON M/S.

### 2.3. One-pot synthesis of GSH-CDS

Glutathione-passivated carbon dots were synthesized by a one-step hydrothermal method. Briefly, 0.350 g prickly pear cactus juice was added to a 1 : 1 solution of water : ethanol (v/v). To the above reaction mixture, 0.300 g of glutathione was added and refluxed for 1 h. The final reaction mixture was packed into a Teflon-lined autoclave and heated at 180 °C for a period of 12 h. The obtained brownish product was cooled to room temperature naturally, and then larger nanoparticles are removed by filtration using a 0.22 μm filter membrane. Finally, the suspension was centrifuged at 12 000 rpm for about 30 min to obtain a yellowish supernatant as a final product. It was then refrigerated at 4 °C for further characterization and analysis.

### 2.4. Detection of As^3+^/ClO^−^ ions with GSH-CDs

As^3+^ ions were detected in optimum conditions at room temperature in tris–HAc buffer medium. Typically, 10 μL GSH-CDs was added into 1000 μL of tris–HAc buffer (10 mM, pH 7.4). The required concentrations of As^3+^/ClO^−^ (0–30 nM/0–200 μM) ions were prepared from the stock solution *via* 20 μL used for the analysis. The sample was diluted with drinking water to obtain a final volume of 3 mL. The reaction mixture of As^3+^ ions was incubated for 2 min at room temperature and then the emission intensity was analyzed, where the response of ClO^−^ ions was recorded immediately. The fluorescence quenching intensity of the GSH-CDs was recorded to identify the selectivity and sensitivity for As^3+^/ClO^−^ ions in the drinking water.

### 2.5. Detection of As^3+^/ClO^−^ ions in real samples

The environmental water samples, including tap, pond, river, and industrial waste water, were collected from various sources near our laboratory and filtered through a 0.22 μm filter to remove the solid impurities. The purified water (100 μL) was mixed with tris–HAc buffer (1000 μL) and then the preferred concentration of As^3+^/ClO^−^ ions was spiked into the above mixture, as shown in Tables 3 and 4. GSH-CDs (10 μL) were added into the above reaction mixture, which was then further diluted with water to obtain a final volume of 3 mL. The fluorescence quenching intensity of GSH-CDs was recorded.

**Table 1:** Various fluorescent sensors for the detection of As^3+^

Materials	Readout mechanism	Working range (M)	LOD	Ref. no.
CdTe QD	Turn off	2 nM to 0.5 μM	2 nM	55
Thiol/CQDs	Turn off	5–100 ppb	0.086 ppb	56
CdTe QD/GSH	Turn off	5 × 10^−6^ to 25 × 10^−5^	2 × 10^−8^	57
CdTe/ZnS	Turn on	1 × 10^−11^ to 1 × 10^−6^	1.3 × 10^−12^	58
GSH/CQDs	Turn off	2–25 × 10^−9^	2.3 × 10^−9^	Present work

**Table 2:** Various fluorescent sensors for the detection of ClO^−^

Materials	Readout mechanism	Working range (M)	LOD (M)	Ref. no.
SiQDs	Turn off	0.01 × 10^−6^ to 50 × 10^−6^	0.01 × 10^−6^	59
Glucose-GCDs	Turn off	0.5 × 10^−6^ to 1000 × 10^−6^	0.3 × 10^−6^	60
Citric acid, urea CDs	Turn off	2 × 10^−6^ to 200 × 10^−6^	2 × 10^−6^	61
MoS_2_ QDs	Turn off	5 × 10^−6^ to 500 × 10^−6^	0.5 × 10^−6^	62
Ethylenediamine, citric acid CDs	Turn off	10 × 10^−6^ to 140 × 10^−6^	4 × 10^−6^	63
GSH/CDs	Turn off	10 × 10^−6^ to 200 × 10^−6^	0.016 × 10^−6^	Present work

**Table 3:** Detection of As^3+^ in environmental water samples

Samples	Spiked [As^3+^]/(nM)	Found As^3+^/(nM)	Recovery (%)	RSD (%)
Tap water	3	2.97	99	1.12
River water	8	7.89	98.62	1.32
Pond water	13	12.95	99.62	2.17
Industrial wastewater	18	18.13	100.72	1.18

**Table 4:** Detection of ClO^−^ in environmental water samples

Samples	Spiked [ClO^−^]/(μM)	Found ClO^−^/(μM)	Recovery (%)	RSD (%)
Tap water	5	5.02	100.4	2.14
River water	10	9.97	99.67	1.56
Pond water	15	14.83	98.92	1.36
Industrial waste water	20	19.77	98.84	2.1

### 2.6. Quantum yield determination

The quantum yield (QY) of the surface-passivated GSH-CDs were investigated with an aqueous solution of quinine sulfate in 0.1 M H_2_SO_4_ as a reference dye, with a QY of 0.54% obtained at an excitation wavelength of 370 nm. The integrated intensities and absorbance of GSH-CDs were compared with quinine sulfate to measure the quantum yield following equation:^50^*Q*_*x*_ = *Q*_std_(*I*_*x*_/*I*_std_)(*η*_*x*_^2^/*η*_std_^2^) (*A*_std_/*A*_*x*_)where, *Q*_*x*_ and *Q*_std_ are the quantum yields of GSH-CDs and quinine sulfate; *η*_*x*_^2^ and *η*_std_^2^ are he refractive indexes of water and H_2_SO_4_; *I*_*x*_ and *I*_std_ are the integrated emission area; and *A*_*x*_ and *A*_std_ are their optical densities of GSH-CDs and quinine sulfate dye. The quantum yield of GSH-CDs was measured to be 12.7%.

## 3. Results and discussion

**Fig. 1:**
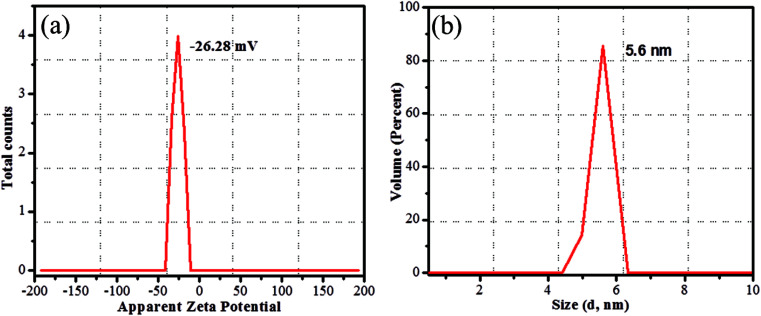
Zeta potential (a) charge of GSH-CDs, (b) hydrodynamic diameter of GSH-CDs.

**Fig. 2:**
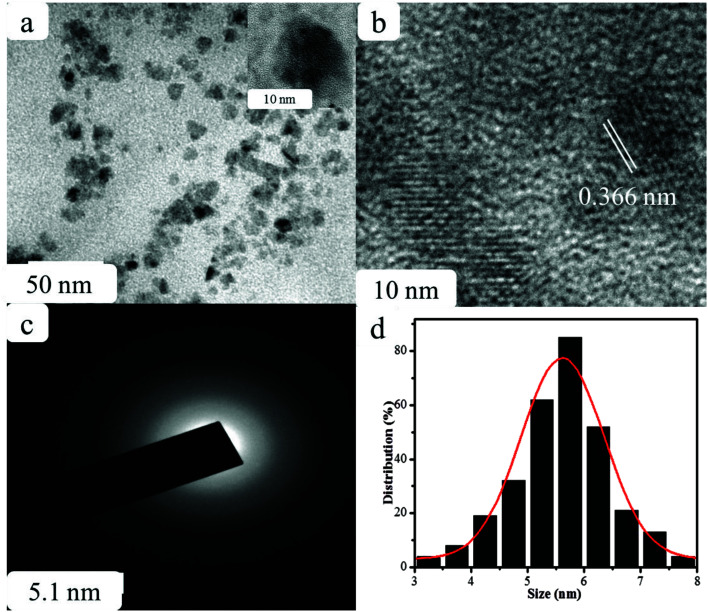
(a) TEM, (b) HR-TEM images, (c) SAED pattern, and (d) particle size distribution histogram of GSH-CDs.

The CDs were synthesized from prickly pear cactus fruits by a one-pot hydrothermal procedure. Since, the prickly pear cactus encloses numerous organic acids (malonic, glutaric, malic, citric, phobic, and piscidic acids) and amino acids (lysine, histidine, arginine, valine, methionine, isoleucine, leucine, and phenylalanine) it was used as a source of carbon.^51^ The glutathione precursor was selectively used to passivate the CDs because of its multiple functional groups (−COOH, –CO, and –NH_2_), which provide an abundance of oxygen- and nitrogen-rich species at their surface. The surfaces of the GSH-CDs were negatively charged due to the passivation of carboxylic, carbonyl and hydroxyl groups. These functional groups act as a sensing probe and have a high ability to chelate with As^3+^/ClO^−^ ions in drinking water samples. The preparation method holds novelty in the surface modification of CDs *via* a single-step green synthesis procedure and in the selective analysis of dual ions. The zeta potential measurement of GSH-CDs the resulted in a negatively charged surface (−26.28 mV) due to the functionalization of the carboxylic group over the surface, as shown in Fig. 1a. The sizes of the GSH-CDs were measured to be 5.6 nm, as shown in Fig. 1b, which is similar to the histogram chart in Fig. 2d.

**Fig. 3:**
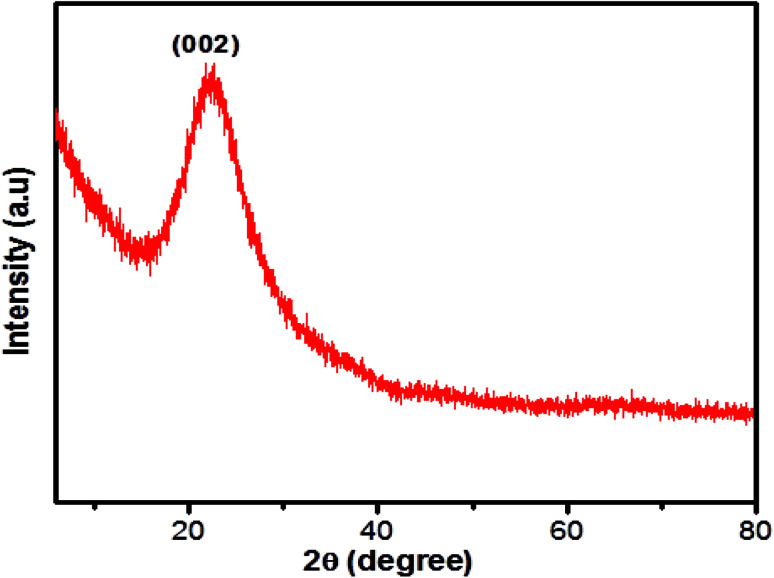
XRD pattern of GSH-CDs.

The structural morphology of the GSH-CDs particles was confirmed by high-resolution transmission electron microscopy (HR-TEM) analysis, as shown in Fig. 2. The TEM images of GSH-CDs found they were dispersed and the particle sizes were in the range of 3.0 to 8.0 nm in histogram chart with an average size of 5.6 nm. The phase purity of the prepared GSH-CDs probe was determined by an X-ray (XRD) diffraction technique. Fig. 3 depicts the XRD profile of the blue-emitting CDs, where a broad peak centered at 22.3° can be recognized as the (002) diffraction pattern, which also endorsed the existence of oxygen-containing functional groups.^52^ The SAED pattern in Fig. 2c indicates the poor crystalline nature. The HR-TEM image of GSH-CDs (Fig. 2b) shows a fringe spacing of 0.366 nm, which agreed well with the spacing of the (002) diffraction pattern.

**Fig. 4:**
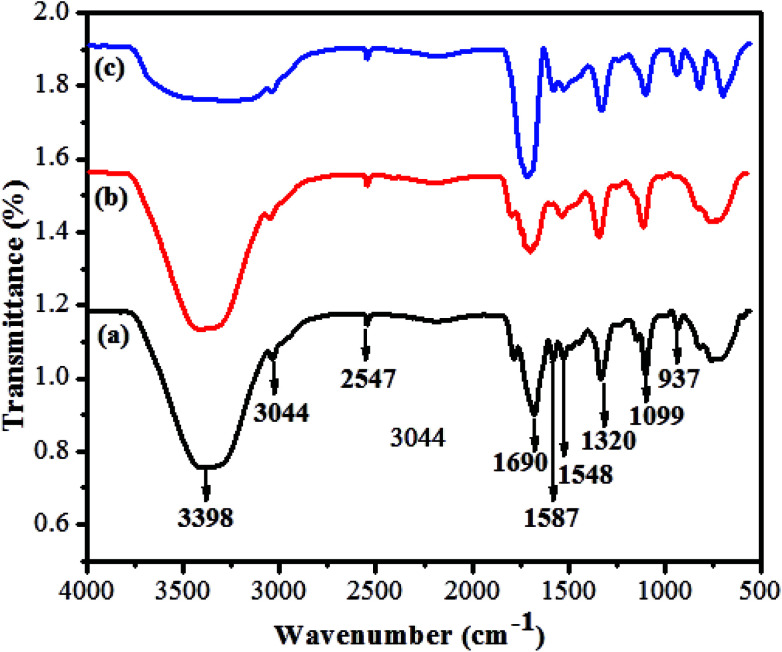
(a) FTIR patterns of GSH-CDs, (b) with As^3+^ and (c) with ClO^−^ ions.

The prepared quantum dots were further confirmed by FT-IR spectroscopy. The functional groups of GSH-CDs were successfully passivated at the surface of the sensor. As shown in Fig. 4a, the broad absorption band at 3398 cm^−1^ was assigned to the stretching vibration of an O–H group and the absorption band at 3044 cm^−1^ was attributed to stretching vibrations of a C–H group. The characteristic absorption bands at 1690 and 937 cm^−1^ were attributed to stretching and bending vibrations of the C–O and O–H groups of carboxylic acid. Furthermore, the absorption bands at 1587 and 1548 cm^−1^ were assigned to stretching and bending vibrations of C=O and NH_2_, respectively. The peaks about 1320 and 2547 cm^−1^ were attributed to stretching of C–O groups and band vibrations of S–H groups in the GSH-CDs. In Fig. 4b, the asymmetric and symmetric stretching vibration intensities at 1690 and 937 cm^−1^ were decreased due to the chelation of COO^−^ ions with As^3+^ ions. In Fig. 4c, the absorption bands intensities at 3398 cm^−1^ and 1032 cm^−1^ were decreased due to reduction of the O–H stretching peaks. New peaks appear in a range of 1000 to 400 cm^−1^ and represent the absorption bands due to the coordination of ClO^−^ ions.

**Fig. 5:**
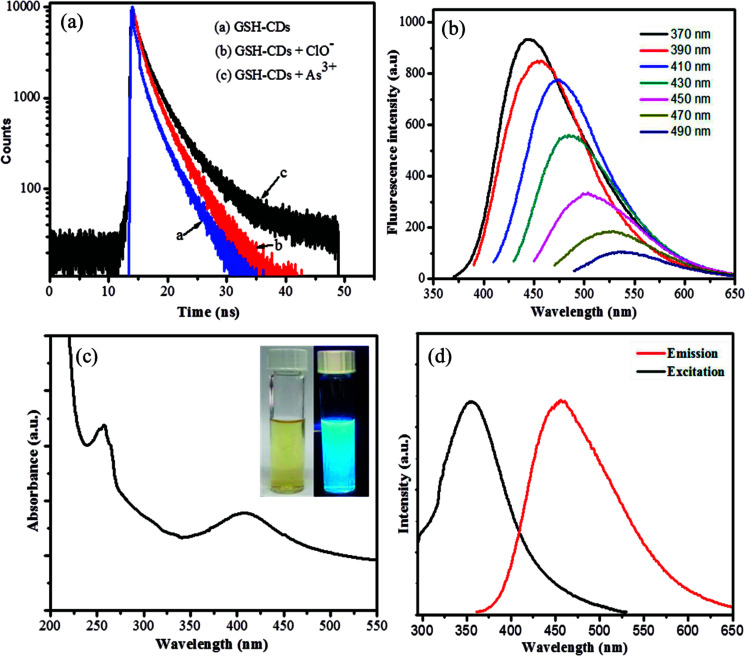
(a) Fluorescence decay curves of GSH-CDs (blue colored curve (a)), GSH-CDs with ClO- (red colored curve (b)), and GSH-CDs with As^3+^ (black colored curve (c)). (b) Excitation-dependent fluorescence emission of GSH-CDs with excitation from 370 to 490 nm. (c) UV-vis absorption spectra of GSH-CDs. The inset image is an aqueous dispersion of GSH-CDs in day light and UV light, respectively. (d) Fluorescence excitation and emission response of the GSH-CDs.

The fluorescence decay of GSH-CDs with and without As^3+^/ClO^−^ ions were measured, as interpreted in Fig. 5a. The quenching mechanism of GSH-CDs were analyzed with the desired concentration of As^3+^/ClO^−^ ions by measuring the correlated charge transfer and excited recombination process in the presence and absence of As^3+^/ClO^−^ ions. The lifetimes of the bare GSH-CDs were found to be very short, as represented by the blue line in the figure, which implies a fast excited recombination process with an average lifetime of 2.752 ns. Furthermore, the decay processes examined with the addition of As^3+^ ions (black line) and ClO^−^ (red line) indicated an increase in the decay component, which holds with an average lifetime of 3.0653 ns and 2.894 ns. These results demonstrated that the quenching of the fluorescence intensity upon the addition of As^3+^/ClO^−^ was a static quenching process.

The emission spectra of the surface-passivated GSH-CDs were observed at different excitation wavelengths in the range of 370 to 490 nm, as shown in Fig. 5b. The obtained spectra signified a red-shift with a decrease in the fluorescence intensity (446–536 nm) by increasing the excitation from 370 to 490 nm, due to the surface energy traps of CDs by the passivation of glutathione. The relative fluorescence quantum yield of oxygen-rich GSH-CDs were measured to be 12.7%. The specificity in quenching of the emission intensity could be used as a signaling unit to identify the presence of toxic ions.

The surface-passivated oxygen-rich CDs were excellently water soluble. UV-vis absorption analysis showed strong absorption bands centered at 278 nm and 402 nm, which were assigned as n–π* transition of C=O bonds and π–π* transition of C=C groups, as shown in Fig. 5c. The emission intensity of the as-prepared GSH-CDs was monitored with a fluorescence spectrophotometer. The blue emission of oxygen-rich CDs revealed a strong emission peak centered at 446 nm with an excitation of 355 nm, as shown in Fig. 5d. The obtained yellow suspension appeared bright blue under UV-visible light (365 nm), as shown in the inset image of Fig. 5d. The fluorescence characterization results showed it was a positive approach to use GSH-CDs as a sensing system to analyze toxic ions.

### 3.1. Influence of pH

**Fig. 6:**
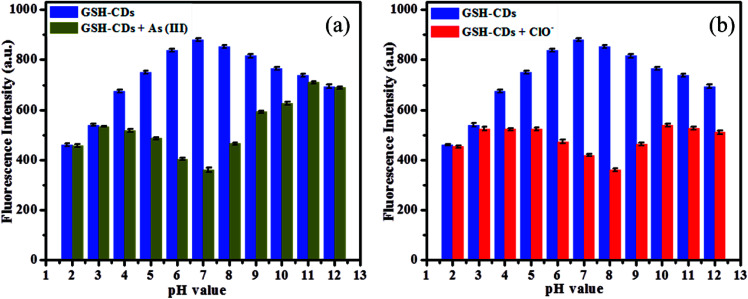
(a) Fluorescence intensity of GSH-CDs (10 μL) at various pH (2–12) with and without As^3+^ (25 nM), and (b) Fluorescence intensity of GSH-CDs at various pH (2–12) with and without ClO^−^ (100 μM).

The fluorescence response of GSH-CDs was investigated under a wide range of pH (2–11) in an aquatic medium, as depicted in Fig. 6a. The functionalized hydroxyl and organic acid groups on CDs were protonated in an acidic medium until the pH was <4, resulting in a low quenching efficiency. As the pH increased from 4 to 8, the fluorescence intensity significantly decreased due to the high quenching efficiency and maximum quenching was observed in a neutral medium (pH = 7). This is due to deprotonation of the functionalized groups, which increases the covalent bond strength between the GSH-CDs and As^3+^ ions. As the pH further increases (pH > 8), the As^3+^ ions tend to form complexes about OH^−^ ions in the aquatic medium, which results in a poor interaction with the CDs.^53^ The maximum response in neutral medium could be attributed to a better interaction between the functionalized CDs and the metal ions. Hence the sensitive and selective detection of As^3+^ ions was feasible in the drinking water sample.

The fluorescence intensity of the GSH-CDs was explored for their use as a suitable sensing probe for the detection of ClO^−^ ions in aqueous solution. The sensitivity of the sensing probe was investigated in a wide range of pH values from 2 to 12 to identify the better response of GSH-CDs, as shown in Fig. 6b. The results revealed that the pH of the medium is an important factor to analyze the correlation between the sensitivity of the probe and the ClO^−^ ions. The pH plays a critical role in the sensing of ClO^−^ ions, whereby the hydroxyl and organic acid groups on the surface of GSH-CDs were protonated in the acid medium and deprotonated in a strong basic medium. The fluorescence intensity varied at different pH values, depending on the oxidative interaction of ClO^−^ ions with the sensing probe. In acidic conditions, the sensitivity of the probe was very poor due to the low interaction with ClO^−^ ions as they were instead converted into HClO. Whereas in alkaline medium at pH > 8, the sensitivity was poor due to the prolonged contact time of ClO^−^ with the sensing probe, leading to its poor efficiency at interacting with the surface functional groups.^54^ Furthermore, NaClO may be hydrolyzed to HClO and ClO^−^ in an aqueous solution and the reaction remains incomplete at pH 6.5 to 8.5. Thus the active HClO and ClO^−^ ions are found to exist in neutral and weakly alkaline media. The fluorescence emission spectra were analyzed for the detection of ClO^−^ ions and the sensor showed a better response at pH values between 6 and 9. The maximum quenching response was observed at pH 8 with the peak centered at 456 nm and excited at 355 nm.

### 3.2. Sensitivity of GSH-CDs toward As^3+^/ClO^−^ ions

**Fig. 7:**
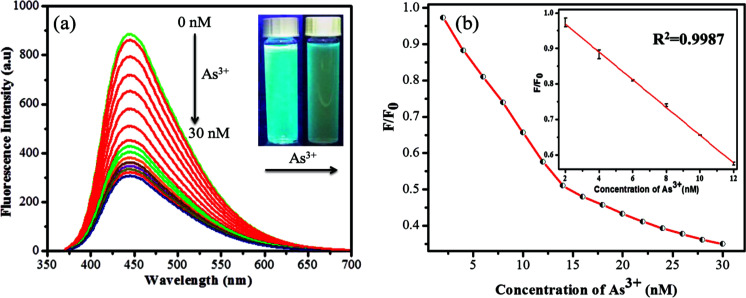
(a) Fluorescence emission response of 10 μL GSH-CDs, to various molar equivalents of As^3+^ ions (0 to 30 nM) in 1000 μL of tris–HAc buffer (10 mM, pH 7.4). (b) Stern–Volmer plot with an inset plot shows the linearity for As^3+^ ions (2 to 12 nM).

The sensitivity study of the emission spectra of the GSH-CDs provided a useful correlation between the quenched emission intensity of the sensing system and the concentration of As^3+^/ClO^−^ ions. The glutathione-passivated CDs showed a high affinity for the effective detection of As^3+^ ions under optimum conditions. As shown in Fig. 7a, upon increasing the concentration of As^3+^ (0–30 nM) ions, the emission intensity of GSH-CDs was strongly quenched, with an emission peak centered at 456 nm as excited at 355 nm.^64,65^ The quenching efficiency of the sensor revealed the sensitivity of the surface functional groups (carboxylic groups) toward As^3+^ ions. The quenching efficiency was directly proportional to the concentration of As^3+^ ions and its linear correlation was plotted to identify the working range. The analyzed data was applied in the Stern–Volmer equation to confirm whether the sensing mechanism was dynamic or static: *F*_0_/*F* = 1 + *K*_sv_*C*, where, *K*_sv_ is a Stern–Volmer constant, *C* is the concentration of metal ions, and *F* and *F*_0_ represent the fluorescence intensity of GSH-CDs in the presence and absence of As^3+^ ions. The plot (Fig. 7b) displays a linear fit (*R*^2^ = 0.9987) when increasing the As^3+^ concentration (2–12 nM) and achieved a lower detection limit of 2.3 nM as calculated by the 3*s*/*k* method (where *s* is the standard deviation of the blank measurement (0.00483) and *k* is the slope (6.19878), so the calculated LOD is = (3 × 0.00483)/(6.19878) = 2.3375).^66^ The achieved detection limit is compared with the previously reported materials for As^3+^ sensing in Table 1, and the results clearly suggest that the GSH-CDs have remarkable sensitivity and indicate the possibility for the probe to be used for As^3+^ sensing in drinking water.

**Fig. 8:**
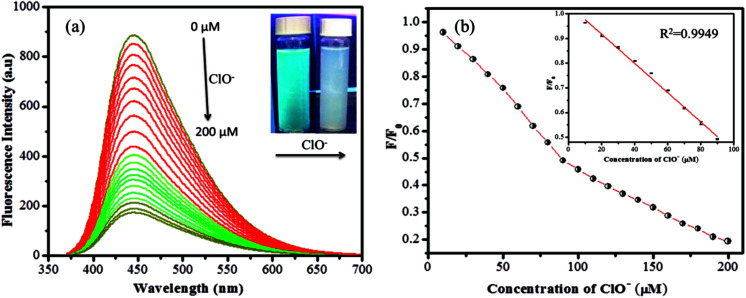
(a) Fluorescence emission response of 10 μL GSH-CDs, to various molar equivalents of ClO^−^ ions (0 to 200 μM) in 1000 μL of tris–HAc buffer (10 mM, pH 7.4). (b) Stern–Volmer plot shows the linearity for ClO^−^ ions (10 to 90 μM).

Similarly the fluorescence quenching of GSH-CDs in the presence of ClO^−^ ions was analyzed in the range of 0–200 μM, as shown in Fig. 8. The sensitivity of the sensing probe revealed an excellent linearity in the range of 10–90 μM with a correlation coefficient of *R*^2^ = 0.9949, as shown in the inset of Fig. 8b. Similar experimental conditions were followed to calculate the detection limit of ClO^−^ ions and it was found to be 0.016 μM. The sensing probe of GSH-CDs showed a strong affinity with ClO^−^ ions in the drinking water. The obtained results are compared with the reported literature in Table 2. The sensitivity analysis suggested that the GSH-CDs can be used as an excellent sensor for As^3+^/ClO^−^ ions with improved performance and simplicity.

### 3.3. Selectivity and coexisting ions effect on GSH-CDs

**Fig. 9:**
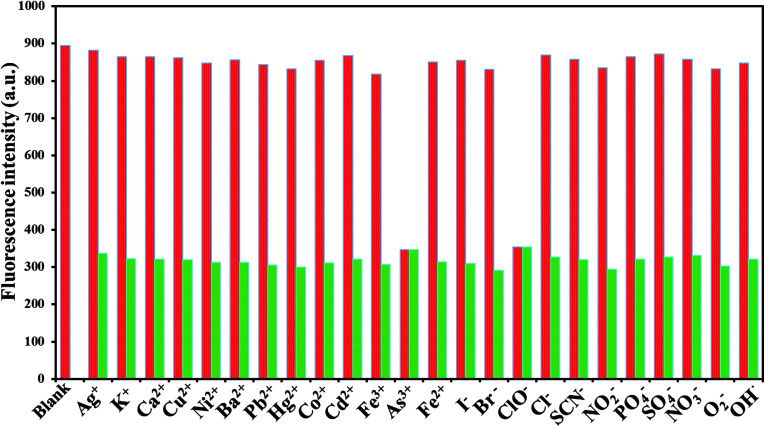
Fluorescence response of 10 μL GSH-CDs, to various molar equivalents of cations (25 nM) and anions (100 μM) in 1000 μL of tris–HAc buffer (10 mM, pH 7.4).

To explore the selectivity of the established fluorescent sensor (GSH-CDs), it was important to perform the analysis under optimized conditions. The fluorescence quenching intensity was studied for As^3+^ (25 nM) and ClO^−^ (100 μM) ions in the presence of other cations (25 nM) and anions (100 μM) (Ag^+^, K^+^, Ca^2+^, Cu^2+^, Ni^2+^, Ba^2+^, Pb^2+^, Hg^2+^, Cd^2+^, Co^2+^, Fe^2+^, Fe^3+^, ClO^−^, Br^−^, I^−^, SCN^−^, NO_2_^−^, PO_4_^3−^, H_2_PO_4_^−^, and SO_4_^2−^, ONOO^−^, ·OH, O_2_^−^˙) in a tris–HAc buffer solution (10 mM, pH 7.4) and 10 μL GSH-CDs solution, as shown in Fig. 9. The fluorescence intensity of the GSH-CDs in the absence and presence of the other metal ions was observed and the change in emission intensity was denoted by a red and green bar.^67^ The drastic quenching efficiency was observed in the presence of As^3+^ or ClO^−^ ions as represented by the green bar. In contrast, no tremendous decrease in emission intensity was observed upon the addition of other metal ions into the GSH-CDs dispersion, as represented by the red bar. These results indicated that the GSH-CDs were predominantly selective toward As^3+^and ClO^−^ ions.

**Fig. 10:**
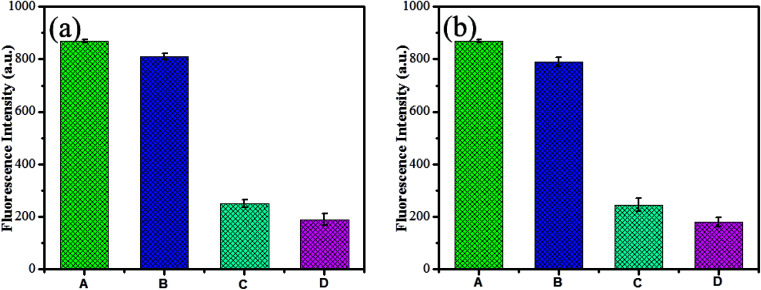
(a) Fluorescence response of (A) 10 μL GSH-CDs, (B) other ionic mixture (Ag^+^, K^+^, Ca^2+^, Cu^2+^, Ni^2+^, Ba^2+^, Pb^2+^, Hg^2+^, Cd^2+^, Co^2+^, Fe^2+^, Fe^3+^, As^3+^, Cl^−^ of 25 nM, and ClO^−^, Br^−^, I^−^, SCN^−^, NO_2_^−^, PO_4_^3−^, H_2_PO_4_^−^, SO_4_^2−^, ONOO^−^, ·OH, and O_2_˙^−^ of 100 μM) without As^3+^ and ClO^−^ ions, (C) other ionic mixtures with 25 nM of As^3+^ and without ClO^−^ ions and (D) other ionic mixtures with 100 μM of ClO^−^ ions of 100 μM and without As^3+^. (b) Is a similar analysis of (a), followed with a higher concentration of other competitive cations (30 nM), and anions (150 μM).

To examine the selectivity of GSH-CDs, the emission responses in the presence of other competitive metal ions were investigated. Under optimum conditions, As^3+^ (25 nM) or ClO^−^ (100 μM) solution were added to a GSH-CDs dispersion of competitive ions. The interference with other ions is depicted in the emission spectra, as shown in Fig. 10a. The emission intensity for As^3+^ or ClO^−^ ions was found to be quenched as they form a complex with the sensing probe. Similarly the quenching efficiency was investigated at a higher concentration of other competitive cations (30 nM) and anions (150 μM), as shown in Fig. 10b. The influence of other competitive ions had an insignificant effect on the emission band, even at higher concentration. This investigation implied that the GSH-CDs as a fluorometric sensor were highly selective and specific to As^3+^or ClO^−^ ions over the other metal ions, indicating the possibility of the sensor to be applied in the sensing As^3+^ or ClO^−^ ions in drinking water.

**Fig. 11:**
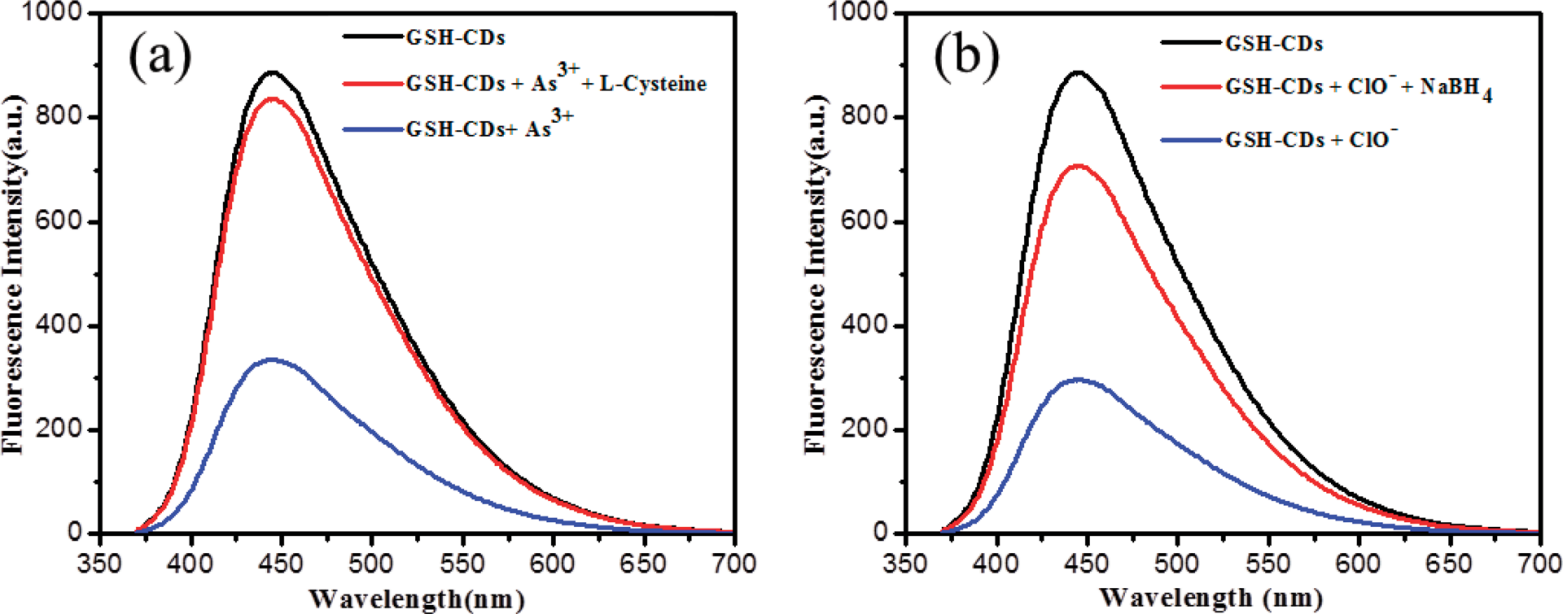
(a) Fluorescence response 10 μL of GSH-CDs for As^3+^ ions (25 nM) and recovery of l-cysteine. (b) Fluorescence response of GSH-CDs for ClO^−^ ions (100 μM) and the recovery of NaBH_4_.

The interference study reveals that the proposed sensor was more selective toward As^3+^or ClO^−^ ions with the coexistence of other metal ions. Yet, it is to be noted that ClO^−^ ions showed a strong interference with GSH-CDs to quench the emission intensity more in the detection of As^3+^ ions. In the attempt to sense ClO^−^ ions, the quenching efficiency of the sensor was significantly altered by interference with As^3+^ ions. This was attributes to the mutual interference of As^3+^ and ClO^−^ ions with the sensing probe. Hence, we focused on the recovery study to prove the selectivity of As^3+^ and ClO^−^ ions. l-Cysteine and sodium borohydride were used as chelators to capture the As^3+^ and ClO^−^ ions as metal chelates. The interference with As^3+^ and ClO^−^ ions toward the GSH-CDs probes were insignificant in the presence of l-cysteine and sodium borohydride, as shown in Fig. 11. The selectivity and specificity for As^3+^ or ClO^−^ ions were due to the stronger affinity with carboxylic and hydroxyl functional groups on the surface of CDs than the other ions.

### 3.4. Sensing mechanism for the detection of As^3+^/ClO^−^ ions

**Scheme 1:**
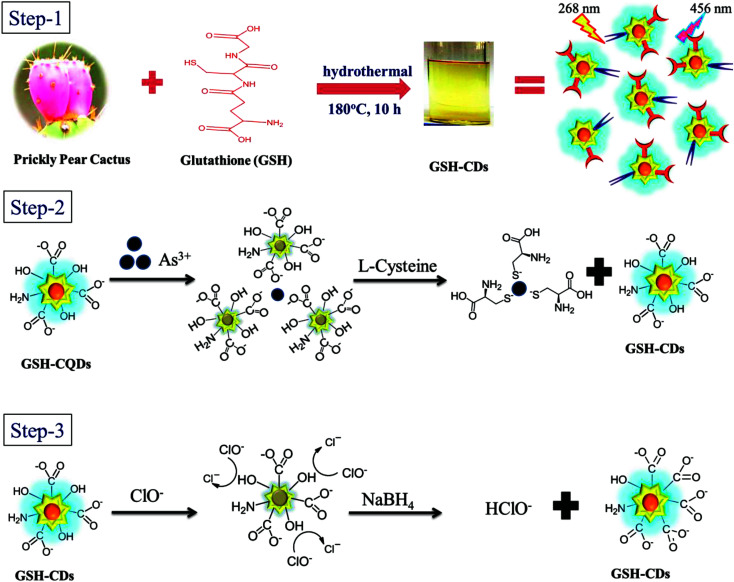
Schematic illustration of the design and working principle.

The fluorescence signal of the GSH-CDs was selectively quenched toward As^3+^/ClO^−^ ions, possibly due to the complex formation of As–O on the surface of the CDs, as represented in Scheme 1. The surface passivation with glutathione enriches the surface with functional groups containing O and N species, which shows a good ability of chelation with ions.^68^ The GSH-CDs showed a high selectivity and sensitivity for As^3+^ ions based on quenched fluorescence *via* a turn-off mechanism, which could be recognized by an inner filter effect, the available functional groups, ion binding interactions, and electron-transfer process.^68^ In the selectivity study, fluorescence spectra were recorded for various metal ions and it was noted that As^3+^ ions had a strong affinity to quenching the fluorescence intensity of GSH-CDs with emission peaks at 456 nm excited at 355 nm. The result showed that As^3+^ ion were more specific toward the organic acid groups of the sensing probe than the other metal ions in this sensing system. Thus the fluorescence quenching may be attributed to the selective interaction between excited GSH-CDs and As^3+^ ions, which could be recognized as an energy or electron-transfer process. The FT-IR spectra confirmed the surface functionalization of organic acid. The aqueous solution of GSH-CDs was strongly quenched by As^3+^ ions in a non-radiative electron-transfer process.

We explored the feasibility of performing the sensitive detection of ClO^−^ ions by GSH-CDs. The sensor exhibited a strong emission peak at 465 nm (*λ*_ex_ = 355 nm) upon titration, whereby the quenching of the fluorescence intensity increased with the increase in the concentration of ClO^−^ ions. The FT-IR spectra confirmed that the hydroxyl functional group on the surface of the GSH-CDs was a reductive group and that it readily interacts with oxidizing substances to quench the fluorescence intensity. The oxidation of hydroxyl groups on the surface brought about a change by ClO^−^ ion with the excellent quenching efficiency of GSH-CDs. The fluorescence quenching mechanism of GSH-CDs with ClO^−^ is proposed in Scheme 1. In the aqueous solution, NaClO hydrolyzes to form HClO and ClO^−^ and this reaction remains incomplete in a pH range of 6.5 to 8.5 and hence both species are found to exist in neutral and weakly alkaline media. An interactive sensing mechanism was thus proposed based on the previously reported literature,^69,70^ whereby:NaOCl + H_2_O → Na^+^ + HClO + OH^−^HClO ↔ H^+^ + ClO^−^

The surface interaction between GSH-CDs and ClO^−^ are discussed on the basis of the redox property. As ClO^−^ is a strong oxidant, it modifies the electron–hole state of oxidized GSH-CDs, whereby:GSH-CDs + ClO^−^ + H_2_O → GSH-CDs˙^+^ + ·Cl + 2OH^−^GSH-CDs + ·Cl → GSH-CDs˙^+^ + Cl^−^

The effective quenching of GSH-CDs was caused by the change in the surface state by the strong oxidizer ClO^−^ ions, which oxidize the surface hydroxyl functional group into –COO^−^. The suggested mechanism below explains the possible quenching ability of CDs with ClO^−^ ions:GSH-CDs-C-OH + ClO^−^→ GSH-CDs-COO^−^ + Cl^−^ + HClO

The interactive mechanism is confirms by the change in surface state *via* quenching the fluorescence intensity of GSH-CDs. The FT-IR spectra evidenced the surface modification of the O–H group. Upon the addition of ClO^−^ ions to the sensing system, the intensity of the O–H stretching peak decreases and a new peak appears at 680 cm^−1^, ascribed to the absorption band of ClO^−^ ions. The organic acid and hydroxyl groups on the surface of GSH-CDs can specifically detect As^3+^/ClO^−^ ions.

### 3.5. Detection of As^3+^and ClO^−^ ions in real samples

**Fig. 12:**
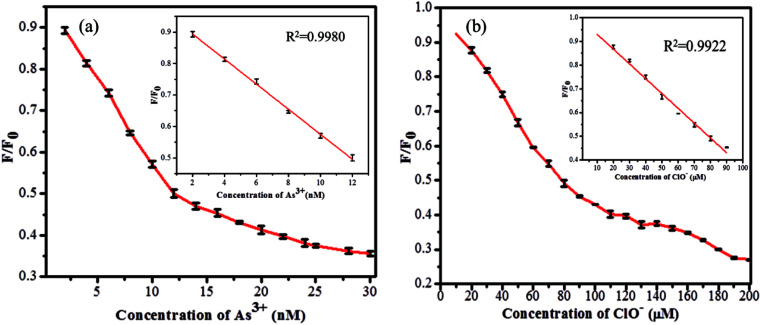
(a) Fluorescence response of GSH-CDs for As^3+^ ions (0–30 nM) in tap water, (b) for ClO^−^ ions (0–200 μM) in tap water. The triplicated data were fitted in the linearity equation of *F*_0_/*F* for tap water sample analysis, shown as in insert image.

To further assess the applicability of the GSH-CDs in practical applications for the detection of As^3+^/ClO^−^ ions, collected real water samples were filtered using a 0.22 μm membrane, and then centrifuged for 10 min at 12 000 rpm. The pH value was altered with tris–HAc buffer solution (pH 7.4) for the sensitive analysis. The optimized tap water sample was spiked with various concentrations of As^3+^and ClO^−^ ions in an increasing range of 0–30 nM and 0–200 μM, respectively. The sensing performance was triplicated and the resulting data were fitted using the linearity equation of *F*/*F*_0_ (Fig. 12). The plot displayed a good linear fits of *R*^2^ = 0.9965 and 0.9922 for As^3+^ and ClO^−^, in a range of 2–12 nM and 10–90 μM, respectively. The fluorescence quenching intensity for the other real samples was recorded and their percentages of recovery are summarized in Tables 3 and 4. The GSH-CDs displayed high selectivity and sensitivity for the As^3+^and ClO^−^ ions, even in the presence of other competing ions. The results clearly demonstrated that the GSH-CDs sensor is applicable for the practical analysis of As^3+^ and ClO^−^ ions in real samples.

## 4. Conclusions

In conclusion, we developed facile, green, and low-cost GSH-CDs through a one-pot hydrothermal route using a prickly pear cactus as a carbonization source for fluorescent CDs. In addition, the surface passivation arises from the effect of glutathione used to improve the surface functional groups, leading to enhanced sensitivity and selectivity. The improved fluorescence signaling performance of CDs *via* an inner filter effect was exploited in the recognition of As^3+^ or ClO^−^ ions. Thus, the quenching of the emission intensity led to a linear response for a detection limit of the assay as low as 2.3 nM for As^3+^ and 0.016 μM for ClO^−^ in drinking water samples. Moreover, the selectivity of the dual sensor was achieved by using l-cysteine and NaBH_4_ as masking agents to recover the As^3+^/ClO^−^ ions. We envisage that this novel assay, with its simplicity, inexpensive nature, water solubility, and photo-stability of the carbon dots, could be used as a fluorescent sensing probe with superior performance over other chemical sensors. The obtained results validated that the GSH-CDs have good potential for real sample analysis.

## Conflicts of interest

There are no conflicts to declare.

## Supplementary Material
